# Ligustrazine-Oleanolic Acid Glycine Derivative, G-TOA, Selectively Inhibited the Proliferation and Induced Apoptosis of Activated HSC-T6 Cells

**DOI:** 10.3390/molecules21111599

**Published:** 2016-11-23

**Authors:** Siling Bi, Fuhao Chu, Mina Wang, Bi Li, Pei Mao, Huazheng Zhang, Penglong Wang, Wenbo Guo, Liang Xu, Liwei Ren, Haimin Lei, Yuzhong Zhang

**Affiliations:** 1 Department of Pathology, Beijing University of Chinese Medicine, Beijing 100102, China; siling2015@163.com (S.B.); wangmina2013@126.com (M.W.); 18811707215@163.com (P.M.); zhanghuazheng1109@163.com (H.Z.); 601354@bucm.edu.cn (L.R.); 2 School of Chinese Pharmacy, Beijing University of Chinese Medicine, Beijing 100102, China; chufhao@163.com (F.C.); libimegan@163.com (B.L.); wpl581@126.com (P.W.); wb_guo@126.com (W.G.); 3 The Second Affiliated Hospital of Shandong University of Chinese Medicine, Jinan 250001, China; bright402@163.com

**Keywords:** ligustrazine-oleanolic acid glycine derivative, selectively inhibition, apoptosis, HSC-T6 cells, hepatic fibrosis

## Abstract

Hepatic fibrosis is a naturally occurring wound-healing reaction, with an imbalance of extracellular matrix (ECM) during tissue repair response, which can further deteriorate to hepatocellular carcinoma without timely treatment. Inhibiting activated hepatic stellate cell (HSC) proliferation and inducing apoptosis are the main methods for the treatment of liver fibrosis. In our previous study, we found that the TOA-glycine derivative (G-TOA) had exhibited more significant inhibitory activity against HepG2 cells and better hydrophilicity than TOA, ligustrazine (TMP), and oleanolic acid (OA). However, inhibiting activated HSC proliferation and inducing apoptosis by G-TOA had not been reported. In this paper, the selective cytotoxicity of G-TOA was evaluated on HSC-T6 cells and L02 cells, and apoptosis mechanisms were explored. It was found that G-TOA could selectively inhibit the proliferation of activated HSC-T6 cells, induce morphological changes, early apoptosis, and mitochondrial membrane potential depolarization, increase intracellular free calcium levels, downregulate the expression of NF-κB/p65 and COX-2 protein, and decrease the ratio of Bcl-2/Bax, thereby inducing HSC-T6 cell apoptosis. Thence, G-TOA might be a potential antifibrosis agent for the therapy of hepatic fibrosis, provided that it exerts anti-fibrosis effects on activated HSC-T6 cells.

## 1. Introduction

Hepatic fibrosis is a naturally occurring wound-healing reaction, characterized by the imbalance of synthesis, degradation, and deposition of extracellular matrix (ECM) during tissue repair response, which can further deteriorate to hepatocellular carcinoma without timely treatment [1,2,3,4]. Chronic hepatitis B and C viral infection, alcoholic abuse, hepatic fat accumulation, biliary obstruction, non-alcoholic steatohepatitis, parasitemia, drug toxicity, inborn errors of metabolism, autoimmune disease, hereditary hemochromatosis, and other factors can cause damage to hepatocytes and elicit inflammatory reaction in the liver [5,6,7]. When liver injury and inflammation reactions are persistent and progressive, the liver cannot regenerate normally and cause fibrosis [2]. However, progressive liver fibrosis can further deteriorate to cirrhosis and ultimately progress to hepatocellular carcinoma without effective treatment. Currently, there is no approved antifibrotic clinical drug.

The inflammation or necrosis of hepatocytes can activate Kupffer cells to release transforming growth factor-β (TGF-β), platelet-derived growth factor (PDGF), tumor necrosis factor-α (TNF-α), reactive oxygen species (ROS), and other soluble agents [1]. These agents can further activate hepatic stellate cells (HSCs), which are quiescent and produce a small amount of ECM in a healthy liver, to cause excessive deposition of ECM in the liver, thus lead to a large number of fibrous dysplasia [8]. As the primary ECM-producing cells in liver, prolonged and repeated activation of stellate cells causes hepatic fibrosis in chronic liver disease [9]. During the regression of hepatic fibrosis, the reduction of activated HSCs is greatly induced by cellular senescence, apoptosis, or the return to the quiescent state [10,11,12]. Therefore, inhibiting activated HSC proliferation and inducing apoptosis are the main methods for the treatment of liver fibrosis.

Oleanolic acid (OA), an oleanane-type triterpenoid that belongs to the pentacyclic triterpene family, is well known for protective activity for the treatment of liver injury, chronic liver fibrosis, cirrhosis, and hepatic carcinoma [13]. In recent reports, due to its favorable properties, OA had been used as a base molecule for further synthetic modifications to develop lead compounds, in particular as potential anti-tumor and anti-fibrotic agents [14,15,16,17,18,19,20,21,22,23]. In the previous report, the lead compound TOA exhibited promising anticancer effects in vitro and in vivo [24,25,26]. It was synthesized by conjugating ligustrazine (TMP) and oleanolic acid (OA), the active ingredients of traditional Chinese medicine (TCM) with promising hepatoprotective activity [27,28,29,30,31]. However, TOA and its structural analogs also demonstrated better inhibitory activity against HSC-T6 than TMP, OA, and other precursor ingredients [32]. The TOA-glycine derivative (G-TOA) had exhibited more significant inhibitory activity against tumor cells than TOA in previous research, which was worthy of further study [33]. However, G-TOA’s inhibition of HSC-T6 proliferation had not been carried out.

In the present study, the selective cytotoxicity of G-TOA was evaluated on rat hepatic stellate cell lines (HSC-T6) and human liver L02 cells by an MTT assay, with colchicine selected as the positive group. The morphology changes of HSC-T6 cells were observed with HE staining. Apoptosis, cell cycle, mitochondrial membrane potential, and intracellular calcium levels were analyzed with the related special fluorescent dyes via flow cytometry. The expression of NF-κB/p65 and COX-2 was detected via immunohistochemical analysis. The expression of Bcl-2 and Bax proteins and ratio of Bcl-2/Bax were detected via Western blot.

## 2. Results

### 2.1. Inhibition of Cell Proliferation

**Figure 1:**
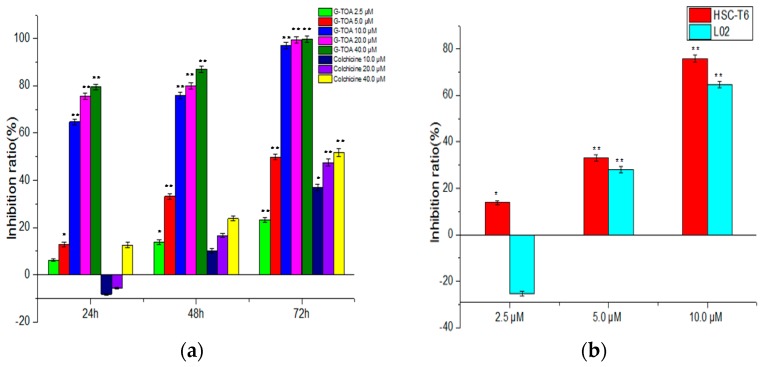
The effect of the TOA-glycine derivative (G-TOA) and colchicine on the proliferation of HSC-T6 cells and L02 cells. (**a**) The effect of G-TOA and colchicine on the proliferation of HSC-T6 cells; (**b**) The effect of G-TOA on the proliferation of HSC-T6 cells and L02 cells were determined for 48 h. Compared with the normal control group. * *p* < 0.05; ** *p* < 0.01.

The G-TOA’s inhibition of HSC-T6 and L02 cell proliferation was evaluated via MTT assay with colchicine selected as the positive group. As shown in Figure 1a, G-TOA could significantly inhibit HSC-T6 cell proliferation in a dose-time dependent manner (IC_50_ = 5.83 ± 0.59 μΜ) and was superior to colchicine. However, the effect of G-TOA on the proliferation of L02 cells was bidirectional regulation. As shown in Figure 1b, it promoted the proliferation at a low concentration (≤2.5 µM) and inhibited at a high concentration (≥5 µM). The results showed that G-TOA could selectively inhibited HSC-T6 cell proliferation.

Combined with the growth curve of HSC-T6 cells and the effect of G-TOA on the morphological changes of HSC-T6 and L02 cells under a light microscope, the appropriate time and doses of administration was selected as 2, 3, and 4 µM for 48 h in order to avoid excessive damage of cells to explore apoptosis mechanisms.

### 2.2. Morphologic Changes

**Figure 2:**
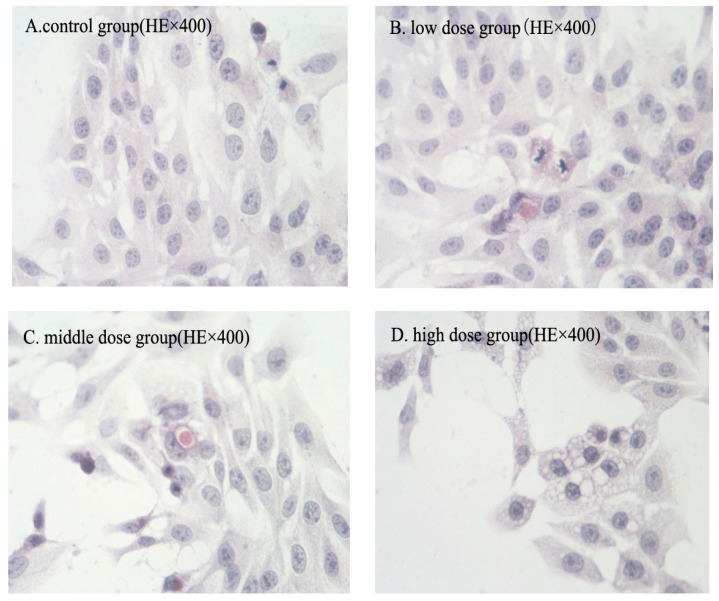
The effect of G-TOA on the morphology of HSC-T6 cells.

The morphologic changes of HSC-T6 cells induced by G-TOA were observed via HE staining. As shown in Figure 2, HSC-T6 cells grown exuberant with morphology intact, cellular junctions tight and arranged in order of control group. After administration, the morphology of HSC-T6 cells was normal, but the extension of cell body was slightly inhibited in the low dose group. Apparent apoptosis features appeared in the middle dose group, including cell shrinkage and rounding, reduction of intercellular connections, nuclear condensation. Moreover, eosinophilic bodies were found in the cytoplasm and the nuclei were stained deeply. In the meantime, the cytoplasm appeared dense. Chromatin undergoes condensation into compact patches against the nuclear envelope in a process known as pyknosis, a hallmark of apoptosis. In the high dose group, normal cell morphology was markedly destroyed, cytoplasm and intercellular junction decreased, and vacuoles appeared. The nucleus broke into several discrete chromatin bodies or nucleosomal units due to the degradation of DNA. Meanwhile, the cells broke apart into multiple vesicles called apoptotic bodies.

### 2.3. Nuclear Fragmentation

**Figure 3:**
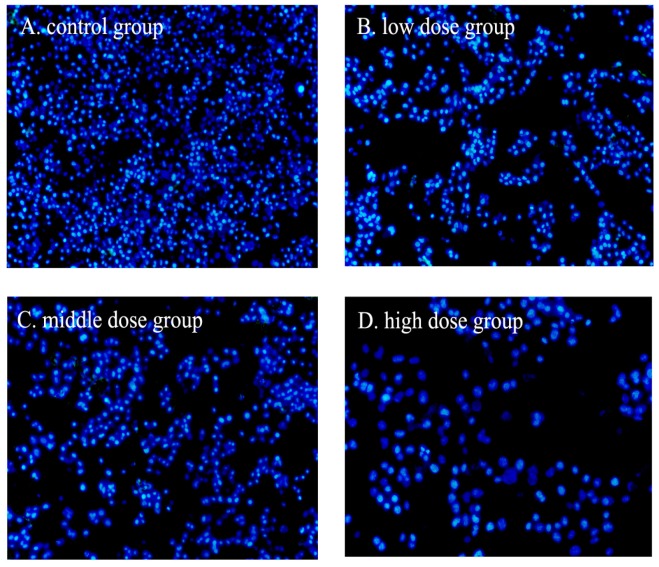
The effect of G-TOA on the nuclear fragmentation of HSC-T6 cells.

Apoptosis could be differentiated from necrosis by their characteristic nuclear changes. The nuclear fragmentation of HSC-T6 cells induced by G-TOA was observed using DAPI staining. As shown in Figure 3, the nuclear staining was slightly blue and homogeneous, and cells with smaller nuclei were rarely seen. After exposure to different concentrations of G-TOA, the contours of some cells became irregular, the nuclei condensed (as brightly blue fluorescence indicated), and apoptotic bodies appeared. The results indicated that G-TOA could induce HSC-T6 cell apoptosis via nuclear fragmentation.

### 2.4. Apoptosis Analysis

**Figure 4:**
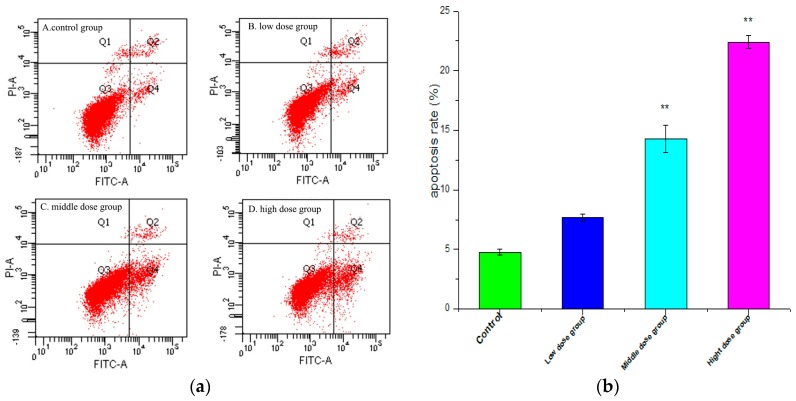
(**a**) The effect of different concentrations of G-TOA on the apoptosis of HSC-T6 cells; (**b**) Apoptosis ratios (including the early and late apoptosis ratios) on different concentrations of administration. Compared with the control group. ** *p* < 0.01.

In an attempt to explicate whether the loss in cell viability induced by G-TOA was associated with apoptosis, the interactions of HSC-T6 cells with G-TOA were further performed via annexin V-FITC/PI double staining. The apoptosis ratios induced by G-TOA on HSC-T6 cells were quantitatively determined using flow cytometry. As shown in Figure 4, apoptosis ratios (including the early and late apoptosis ratios) increased from (7.70 ± 0.290)% to (14.30 ± 1.153)% and (22.40 ± 0.536)%, respectively, while that of the control was only (4.77 ± 0.218)%. The differences were statistically significant (*p* < 0.01). The results indicated that G-TOA could induce apoptosis in HSC-T6 cells.

### 2.5. Cell Cycle Measurement

**Figure 5:**
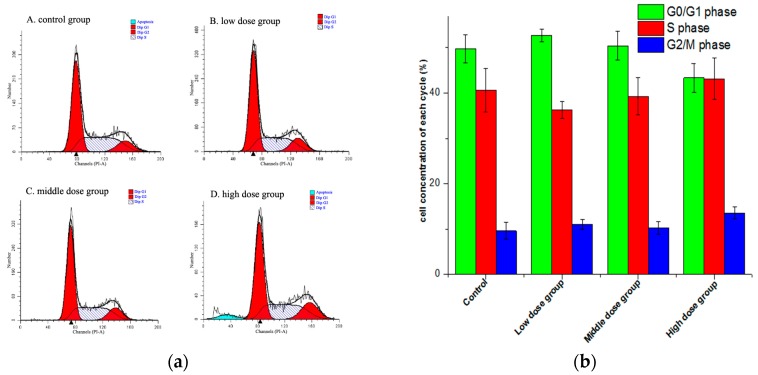
(**a**) The effect of different concentrations of G-TOA on the cell cycle of HSC-T6 cells; (**b**) The ratios of different phases of cell cycle on different concentrations of administration.

Cell cycle was evaluated via propidium iodide (PI) staining using flow cytometry. As shown in Figure 5, the cell percentage in the G0/G1 phase had a smaller increase in the middle dose group, while the proportion in the G1 phase and the G2 phase did not change significantly (*p* > 0.05). With the concentration increased, the percentage in the S phase in the drug intervention group decreased and then increased. However, a sub-diploid apoptosis peak appeared in the high dose group. This indicated that there was no significant effect on the cell cycle of HSC-T6 cells, while the high dose of G-TOA could significantly induce HSC-T6 cell apoptosis.

### 2.6. Changes in Mitochondrial Membrane Potential (∆ψm)

**Figure 6:**
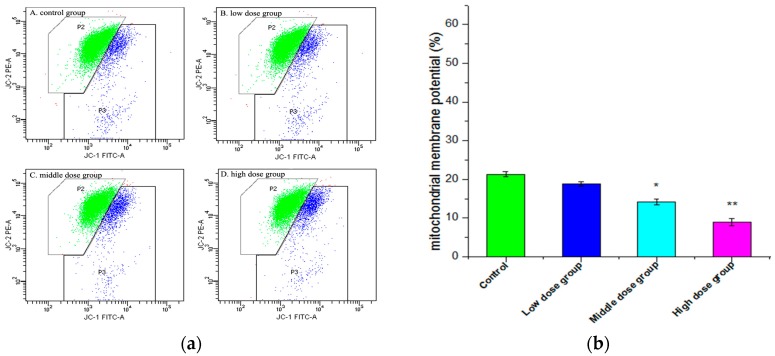
(**a**) The effect of different concentrations of G-TOA on the mitochondrial membrane potential of HSC-T6 cells. (**b**) The green ratios on different concentrations of administration. Compared with the control group. * *p* < 0.05; ** *p* < 0.01.

Depolarization of ΔΨm is a critical step that occurs in all cell types undergoing apoptosis. In this paper, ΔΨm was measured quantitatively by the fluorescent dye JC-1. As shown in Figure 6, the green fluorescent ratio increased from (21.30 ± 0.681)% in the control group to (18.83 ± 0.546)%, (14.15 ± 0.804)%, and (8.97% ± 0.940)%, respectively, with different concentrations of G-TOA. The results indicated that G-TOA could induce mitochondrial membrane potential depolarization and lead to HSC-T6 cell apoptosis.

### 2.7. Intracellular Free Ca^2+^ Detection

**Figure 7:**
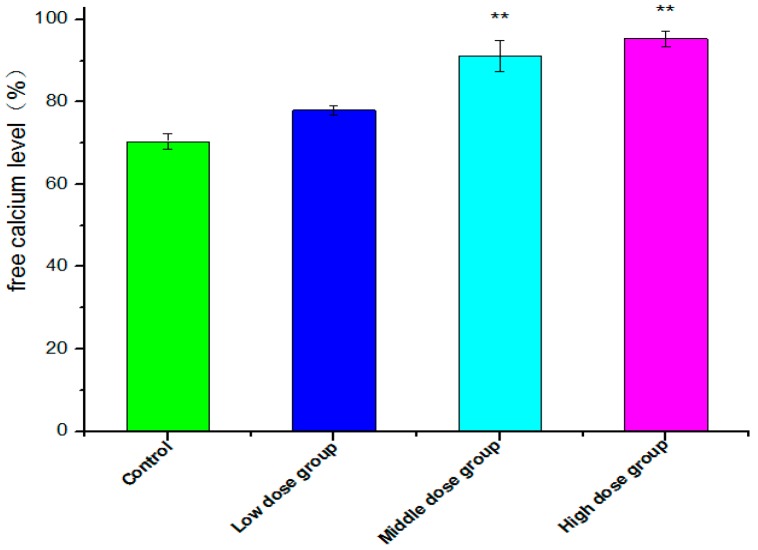
The effect of G-TOA on the free calcium level of HSC-T6 cells. Compared with the control group; ** *p* < 0.01.

A sustained increase in intracellular Ca^2+^ concentrations is recognized as a factor for cell death or injury [34]. The level of intracellular free Ca^2+^ was examined by the fluorescent dye Fluo-3AM, which was converted to Fluo-3 upon deacetylation within the cells, and Fluo-3 increases green fluorescence upon Ca^2+^ binding [35]. As shown in Figure 7, with the concentration of G-TOA increasing from 2.0 μM, 3.0 μM to 4.0 μM, intracellular free Ca^2+^ fluorescence increased dramatically from (77.97 ± 1.128)% to (91.13 ± 3.763)% and then to (95.30 ± 1.840)%. The differences were statistically significant (*p* < 0.01). The results indicate that G-TOA induces HSC-T6 cell apoptosis with the increase in intracellular free Ca^2+^ levels.

### 2.8. Immunohistochemical Analysis

**Figure 8:**
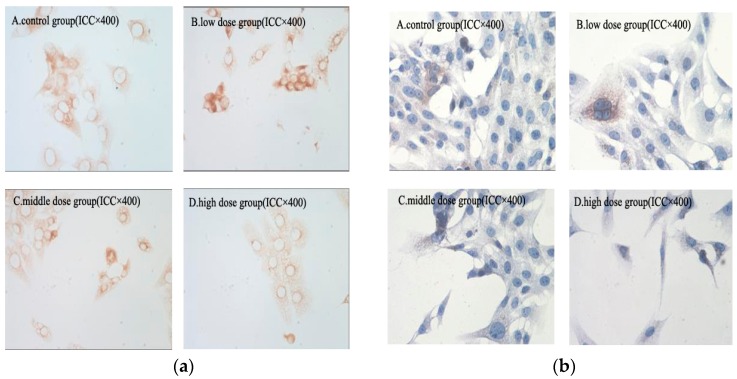
(**a**) The effect of G-TOA on the expression of NF-κB/p65 protein in HSC-T6 cells; (**b**) The effect of G-TOA on the expression of COX-2 protein in HSC-T6 cells.

NF-κB/p65 has been shown to be associated with the inhibition of cell apoptosis [36]. COX-2 can promote the metastasis of cells by promoting tumor angiogenesis and by inhibiting cell apoptosis [37,38]. The expression of NF-κB/p65 and COX-2 protein in HSC-T6 cells was detected via immunohistochemical analysis. As shown in Figure 8, the expression of NF-κB/p65 and COX-2 protein in HSC-T6 cells decreased in a dose-dependent manner compared with the control group. The result revealed that G-TOA could prevent NF-κB activation and suppress expression of COX-2. This indicates that G-TOA might induce HSC-T6 cell apoptosis by suppression of NF-κB/p65 and COX-2 expression.

### 2.9. Expression of Bax, Bcl-2, and the Ratio of Bcl-2/Bax

**Figure 9:**
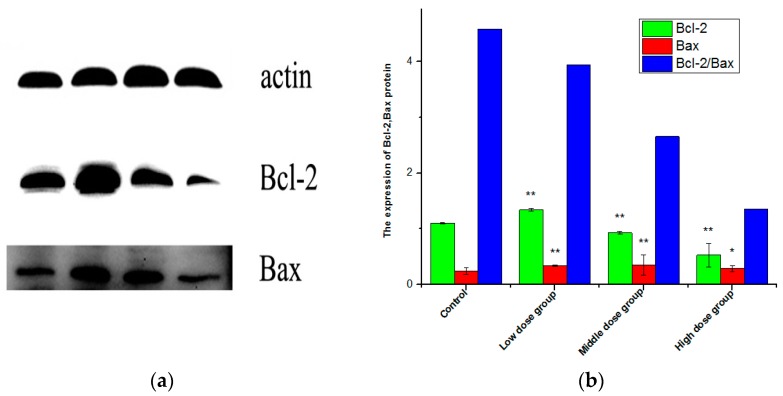
The effect of G-TOA on the expression of Bcl-2 and Bax protein (**a**) and the ratio of Bcl-2/Bax of HSC-T6 cells (**b**) Comparison with the normal control group. * *p* < 0.05; ** *p* < 0.01.

The balance between Bax and Bcl-2 is critical in activating and deactivating cellular apoptotic machinery [39,40]. In this study, the expression of Bax, Bcl-2 proteins, and the ratio of Bcl-2/Bax of HSC-T6 cells induced by G-TOA were determined using Western blot analysis. As shown in Figure 9, compared with the control group, the expression of Bcl-2 protein decreased significantly after different concentrations of administration (*p* < 0.05), while the expression of Bax protein increased, and the ratio of Bcl-2/Bax decreased with increasing concentrations of administration (*p* < 0.05). This indicates that G-TOA can upregulate the expression of Bax and downregulate the expression of Bcl-2 to cause HSC-T6 cell apoptosis.

## 3. Discussion

In recent reports, a large number of oleanolic acid derivatives were designed, synthesized, and evaluated for antitumor activity, in particular as potential anti-hepatic carcinoma and anti-fibrotic agents, which had made outstanding progresses [21,23]. The hepatoprotective activity of OA could be due to its anti-oxidant and anti-inflammatory effects, as well as its effect on drug-metabolizing enzymes [13]. Most of the derivatives could induce hepatocellular carcinoma cell apoptosis through the mitochondrial pathway, the ROS generation, and mitochondrial fatty acid oxidation, along with cell cycle arrest [15]. G-TOA was discovered via the optimization of the ligustrazine-oleanolic acid derivative (TOA), an anticancer and antifibrotic lead compound [24,25,26,32]. Moreover, it had better anti-tumor activity and hydrophilicity [33]. However, the antifibrotic activity of G-TOA has not been reported. HSCs are the major precursor of activated myofibroblasts that can produce ECM proteins during liver fibrosis. Currently, many treatments under evaluation can inhibit either their activation or their proliferation to affect the accumulation of activated HSCs [41]. In the present study, G-TOA could selectively inhibit the proliferation of activated HSC-T6 cells, while there was no significant inhibition against L02 cells at low concentrations. G-TOA can induce HSC-T6 early cell apoptosis, change in normal cellular morphology, and apparent apoptosis features, such as cell shrinkage, nuclear chromatin margination, condensed nuclei, nuclear fragmentation, and the formation of eosinophilic bodies.

Inhibiting activated HSC proliferation and inducing apoptosis are the main approaches for the treatment of liver fibrosis. The initiation of apoptosis is regulated by activation mechanisms, including the intrinsic pathway (the mitochondrial pathway) and the extrinsic pathway [42]. Mitochondrion is considered to play a key and even central role in the apoptotic process [43,44]. Mitochondrial dysfunction can participate in the induction of apoptosis and is suggested to be central to the apoptotic pathway [45]. It has been demonstrated that the opening of the mitochondrial permeability transition pore can induce transmembrane potential depolarization to release the apoptogenic factors generated when cells are stressed from the intermembrane space in the cytosol and from the loss of oxidative phosphorylation. The intrinsic pathway is activated by intracellular signals generated when cells are stressed and depends on the release of proteins from the intermembrane space of mitochondria [42,46,47,48]. The extrinsic pathway is activated by extracellular ligands that can bind to cell-surface death receptors to form the death-inducing signaling complex (DISC) [42]. Moreover, as one of the extrinsic pathways, the increase in intracellular calcium levels induced by the drug can cause apoptosis via calcium binding protease calpain. Calcium is a small signaling molecule regulating various biological cell functions [49]. Ca^2+^ overload plays an important role in the initiation and regulation of cell apoptosis [50] and leads to the activation of pro-apoptotic factors, resulting in apoptosis [51]. The pro-apoptotic effect of Ca^2+^ is mediated by Ca^2+^-sensitive factors. The Ca^2+^ dynamics appear to be modulated by the apoptosis-regulating Bcl-2 family proteins [52]. The members of the Bcl-2 family are important regulators of mitochondrial integrity, mitochondria-initiated cytochrome C release, and caspase activation, including Bax and Bcl-2. Bax is a pro-apoptotic protein that can regulate cytochrome C release from mitochondria under a variety of stress conditions, whereas Bcl-2 is an anti-apoptotic protein that prevents cytochrome C release by heterodimerizing with Bax [53]. In this paper, we found that the expression of Bcl-2 protein decreased significantly after different concentrations of administration (*p* < 0.05), while the expression of Bax protein increased, and the ratio of Bcl-2/Bax decreased, with increasing concentrations of administration (*p* < 0.05). This indicates that G-TOA can upregulate the expression of Bax and downregulate the expression of Bcl-2 to cause HSC-T6 cell apoptosis.

The NF-κB signaling pathway has particular relevance to several liver diseases including hepatitis, liver fibrosis, cirrhosis, and hepatocellular carcinoma, which is a potential target for the development of hepatoprotective agents [54]. NF-κB regulates multiple essential functions in hepatocytes and HSCs [3], and regulates the expression of genes involved in antiapoptosis, proliferation, and metastasis [55]. The NF-κB signaling pathway is related to ECM protein production and upregulates proinflammatory genes encoding cytokines, chemokines, and adhesion molecules in activated HSCs [56]. Associated with apoptosis of HSCs [57], the attenuation of NF-κB activation in HSCs is based on the development of anti-fibrotic drugs and treatments. In addition, NF-κB, by inducing COX-2 expression, may regulate human myofibroblastic HSC proliferation negatively [58]. COX-2, an important enzyme in the starting inflammatory reaction, has been confirmed to contribute to HSC activation [59]. COX-2 can participate in liver fibrosis-mediating liver inflammation and damage, and the blockage of its expression has been associated with antifibrotic activity [60,61,62]. In our previous study, we found that TOA, the precursor of G-TOA, had exerted antitumor activity by preventing the expression of NF-κB/p65 and COX-2 in vivo and in vitro [25,26]. In the present study, we have also found that G-TOA can suppress NF-κB activation and downregulate the expression of COX-2 to suppress HSC proliferation and induce apoptosis.

In summary, our results demonstrate that G-TOA can selectively inhibit the proliferation of activated HSC-T6 cells, cause apoptosis through multiple channels, induce mitochondrial membrane potential depolarization, increase intracellular free calcium levels, downregulate the expression of NF-κB/p65 and COX-2 protein, upregulate the expression of Bax, and downregulate the expression of Bcl-2 to decrease the ratio of Bcl-2/Bax. Therefore, G-TOA is a potential antifibrosis agent for the therapy of hepatic fibrosis, provided that it exerts anti-fibrosis effects on activated HSC-T6 cells. However, it needs to be determined whether G-TOA suppresses the development of liver fibrosis in both animal models and humans.

## 4. Materials and Methods

### 4.1. Inhibition of Cell Proliferation

The HSC-T6 cells and L02 cells were purchased from the Chinese Academy of Medical Sciences Peking Union Medical College. Cells were cultured in DMEM medium supplemented with 10% (*v*/*v*) fetal bovine serum and 1% (*v*/*v*) penicillin-streptomycin (Thermo Technologies, New York, NY, USA) at 37 °C in a humidified atmosphere with 5% CO_2_. The growing cells at a density of 3 × 10^3^ cells/100 μL were exposed to various concentrations of G-TOA and incubated in a 96-well microtiter plate for 72 h (37 °C, 5% CO_2_). After a MTT solution (20 μL/well, 5 mg/mL) was added to each well, the plate was incubated for 4 h. Then, the media was removed. Formazan crystals were dissolved with DMSO (150 μL/well). After shaking for 10 min, the absorbance was quantified at 490 nm with the BIORAD 550 spectrophotometer (Bio-Rad Life Science Development Ltd., Beijing, China). Wells containing no drugs were used as blanks. The IC_50_ values were defined as the concentration of compound that produced the 50% reduction of surviving cells and calculated using the Logit method. The cell growth inhibitory rate was calculated with Equation (1):

(1) $$\mathrm{Inhibition}\;(\%)=(1-\;\frac{\mathrm{OD}\;\mathrm{Administration}-\mathrm{OD}\;\mathrm{Blank}}{\mathrm{OD}\;\mathrm{Control}-\mathrm{OD}\;\mathrm{Blank}})\times 100\%$$

### 4.2. The Morphologic Change

Cells were seeded at a concentration of 3 × 10^4^ cell/mL in 12-well tissue culture plates and pretreated with G-TOA at different concentrations (2, 3, and 4 µM) for 48 h. Cover slips were washed twice with PBS, removed, and treated with acetone for 10 min at −20 °C. Then, cells were washed with PBS and stained with hematoxylin for 3 min. The stained nuclei were rinsed with running water for 1 h followed by eosin staining for another 2 min. Finally, stained sections were dehydrated using gradient ethanol and cleared in xylene [63,64]. The cellular morphology was photographed via light microscopy (Nikon, Kobe, Japan).

### 4.3. DAPI Staining

DAPI staining was performed according to our previous study with minor modifications [22]. In brief, HSC-T6 cells were seeded at a concentration of 3 × 10^4^ cell/mL in 12-well tissue culture plates and pretreated with G-TOA at different concentrations (2, 3, and 4 µM) for 48 h. Then, the culture medium containing compounds was removed and washed with PBS twice, and cells were fixed in ethanol for 10 min and stained with DAPI. After the cells were washed with PBS twice, they were photographed under a fluorescence microscope.

### 4.4. Apoptosis Analysis

An Annexin V-FITC/PI apoptosis detection kit was used according to the manufacturer’s protocol. Briefly, HSC-T6 cells (3 × 10^4^ cells/mL) were treated with G-TOA (2, 3, and 4 µM) for 48 h. Total cells were then washed with cold PBS twice and re-suspended gently in 200 µL of binding buffer. According to the manufacturer’s instructions, Annexin V-FITC and PI (YEASEN, Shanghai, China) were added to each sample. The mixture was incubated for 20 min in a dark place and then analyzed via a BD FACSCanto II fluorescence-activated cell sorter (Becton Dickinson and Company, Franklin Lakes, NJ, USA).

### 4.5. Cell Cycle Measurements

HSC-T6 cells (3 × 10^4^ cells/mL) treated with G-TOA for 48 h were harvested, washed twice in PBS, and exposed to 0.1% Triton X-100 in PBS supplemented with RNA-ase (20 μg/mL) for 30 min at 37 °C. Afterwards, DNA was stained by propidium iodide (50 μg/mL) for 10 min in a dark place and then analyzed by a BD FACSCanto II fluorescence-activated cell sorter. A minimum of 100,000 cells per sample was analyzed at a flow rate of 300 cells·s^−1^.

### 4.6. Intracellular Free Ca^2+^ Detection

The level of intracellular free Ca^2+^ was examined by the fluorescent dye Fluo-3AM. HSC-T6 cells (10 × 10^4^ cells/mL) in a logarithmic growth phase were cultured in 6-well plates for 24 h at 37 °C in an incubator with 5% CO_2_. After the cells were exposed to different concentrations of G-TOA (2, 3, and 4 µM) for 48 h, they were harvested and washed twice with cold PBS, then resuspended in HBSS buffer with 10 μM Fluo-3AM (Shanghai Beyotime Biotech. Co., Ltd., Shanghai, China), and incubated for 30 min at 37 °C in the dark. Detection of intracellular Ca^2+^ was carried out by a flow cytometer at 506 nm excitation wavelength [34,35].

### 4.7. Mitochondrial Membrane Potential Assay

Prepared HSC-T6 cells were washed with PBS and incubated in a medium containing JC-1 dye for 20 min at 37 °C in the dark. The mitochondrial depolarization patterns of the cells were observed by flow cytometry analysis according to the manufacturer’s instructions (Beyotime, Shanghai, China).

### 4.8. Immunohistochemical Staining

HSC-T6 cells were treated with or without G-TOA (2, 3, and 4 µM) for 48 h. Total cells were fixed in 4% formaldehyde for 30 min at 37 °C, and then washed three times with PBS. Subsequently, cells were treated with 2% H_2_O_2_ in methanol for 20 min to block endogenous peroxidase activity, followed by another wash, and then blocked with 10% BSA for 30 min. The cells were incubated with a primary antibody (anti-p65 and COX-2) at 4 °C overnight. The next day, cells were washed three times in PBS and then incubated with a second antibody. After being washed with PBS three times, cells were treated with SABC for 30 min at 37 °C and then developed with DBA. After being counterstained nuclei with hematoxylin, cells were photographed via Olympus IX71 inverted microscopy (Olympus, Tokyo, Japan) with 400× actual magnification.

### 4.9. Expression of Bax, Bcl-2, and the Ratio of Bcl-2/Bax

The cells were harvested following treatment. They were subsequently lysed in a buffer (50 mmol/L Tris–HCl, pH 8.0; 100 mmol/L NaCl; 1 mmol/L EDTA; 1 mmol/L dithiothreitol; 1% Triton X-100; 0.1% sodium dodecyl sulfate; 50 mmol/L sodium fluoride, and 1 mmol/L sodium vanadate) containing a mixture of protease inhibitors. The lysate was incubated on ice for 30 min and then centrifuged at 12,000 rpm for 10 min at 4 °C. The supernatant was collected, and protein concentration was determined using a BCA kit. Equal amounts of protein were fractionated by sodium dodecyl sulfate–polyacrylamide gel electrophoresis and subsequently transferred to a nitrocellulose membrane (Amersham Pharmacia Biotech, London, UK). The membrane was incubated in a fresh blocking buffer (containing Tris-buffered saline, 0.1% Tween-20 in Tris-buffered saline, pH 7.4) at room temperature for 1 h and then incubated with an anti-Bcl-2 antibody and an anti-Bax antibody overnight at 4 °C. The blots were washed three times in TBS-T for 5 min and then incubated with specific peroxidase-coupled secondary antibodies (anti-mouse IgG-HRP or anti-rabbit IgG-HRP). The bound antibodies were visualized using an enhanced chemiluminescent detection system and then exposed to X-ray films (Kodak, Rochester, NY, USA). The images were scanned with a GS800 Densitometer Scanner (Bio-Rad, Hercules, CA, USA), and OD data were analyzed using Quantity One software (Bio-Rad). In these analyses, β-actin was used as an internal reference.

### 4.10. Statistics

Statistical significance was evaluated with a one-way ANOVA, with Bonferroni multi-comparison post-test correction, and with Dunnett’s test using SPSS 17.0 (SPSS Inc., Kraków, Poland). *p*-Values < 0.05 were considered significant, and *p*-values < 0.01 were considered very significant.
